# Four amino acids as serum biomarkers for anti-asthma effects in the ovalbumin-induced asthma mouse model treated with extract of *Asparagus cochinchinensis*

**DOI:** 10.1186/s42826-019-0033-x

**Published:** 2019-12-30

**Authors:** Jun Young Choi, So Hyun Kim, Ji Eun Kim, Ji Won Park, Mi Ju Kang, Hyeon Jun Choi, Su Ji Bae, Jae Ho Lee, Young-Suk Jung, Dae Youn Hwang

**Affiliations:** 1 Department of Biomaterials Science, College of Natural Resources and Life Science, Pusan National University, 50 Cheonghak-ri, Samnangjin-eup Miryang-si, Gyeongsangnam-do 50463 South Korea; 2 College of Pharmacy, Pusan National University, Busan, 46241 South Korea; 3 Wellbeing Product Regional Innovation System Center, Pusan National University, Gyeongsangnam-do, 50463 South Korea

**Keywords:** *Asparagus cochinchinensis*, Fermentation, *Weissella cibaria*, Asthma, Therapeutic effects, Metabolomics, Amino acid

## Abstract

The butanol extract of *Asparagus cochinchinensis* roots fermented with *Weissella cibaria* (BAW) effectively prevents inflammation and remodeling of airway in the ovalbumin (OVA)-induced asthma model. To characterize biomarkers that can predict the anti-asthmatic effects induced by BAW treatment, we measured the alteration of endogenous metabolites in the serum of OVA-induced asthma mice after administration of low concentration BAW (BAWLo, 250 mg/kg) and high concentration BAW (BAWHi, 500 mg/kg) using ^1^H nuclear magnetic resonance (^1^H-NMR) spectral data. The number of immune cells and serum concentration of IgE as well as thickness of the respiratory epithelium and infiltration of inflammatory cells in the airway significantly recovered in the OVA+BAW treated group as compared to the OVA+Vehicle treated group. In the metabolic profile analysis, the pattern recognition showed completely separate clustering of serum analysis parameters between the OVA+Vehicle and OVA+BAW treated groups. Of the total endogenous metabolites, 19 metabolites were upregulated or downregulated in the OVA+Vehicle treated group as compared to the Control treated group. However, only 4 amino acids (alanine, glycine, methionine and tryptophan) were significantly recovered after BAWLo and BAWHi treatment. This study provides the first results pertaining to metabolic changes in the asthma model mice treated with OVA+BAW. Additionally, these findings show that 4 metabolites can be used as one of biomarkers to predict the anti-asthmatic effects.

## Introduction

Asthma, a widespread chronic inflammatory disease of the respiratory airway, is characterized by airway hyper-responsiveness, mucus hypersecretion, infiltration of the airway by eosinophils and T helper 2 (Th2) cells, and airway remodeling [1–3]. Among the numerous physiological events leading to clinical symptoms, bronchoconstriction is the predominant event characterized by contraction of the airway smooth muscles, which is regulated by histamine, tryptase, leukotrienes and prostaglandins released from mast cells [4]. Also, airway hyperresponsiveness is considered a major feature of asthma, and includes inflammation, dysfunctional neuroregulation and structural changes. This response can be effectively reduced by treatment directed towards reducing inflammation [5]. Furthermore, airway remodeling is associated with a progressive loss of lung function. These structural changes are strongly associated with thickening of the sub-basement membrane, sub-epithelial fibrosis, airway smooth muscle hypertrophy and hyperplasia, blood vessel proliferation and dilation, and mucous gland hyperplasia and hypersecretion [6].

To better understand the metabolic phenotypes of asthma, comprehensive analysis of metabolites have been previously investigated in several animal models treated with OVA. Pathological biomarkers related to OVA-sensitized BALB/c mice identified 16 potential metabolites. Especially, 6 metabolites (dodecanoic acid (P1), myristic acid (P2), phytosphingosine (P3), sphinganine (P4), inosine (P13) and taurocholic acid (P15)) were initially considered to have potential relevance in the pathogenesis of OVA-induced mice [7].

Furthermore, several studies have screened the metabolic biomarker in the bronchoalveolar lavage fluid (BALF) and serum of OVA-challenged mice treated with few drugs, to anticipate the anti-asthma effects. Various metabolites in energy metabolism (mannose, arabinose and galactose), amino acid metabolism (5-methoxy-tryptophan and N-acetyl-tyrosine) and lipid metabolism (diglycerides and triglycerides) were significantly altered in the BALF from OVA-challenged BALB/c mice treated with Vehicle or dexamethasone (Dex) [8]. Also, the several plasma metabolites, such as serotonin, C3DC + C4OH, camitine, tyrosine and arginine, increased after OVA challenge but were controlled by fenretinide treatment in A/J mice [9]. Moreover, 12-hydroxy-17,18-epoxyeicosatetraenoic acid (12-OH-17,18-EpETE) was identified as one of the major biosynthesized molecule which was amplified by omega-3 fatty acid eicosapentaenoic acid (EPA) administration in OVA-challenged C57BL/6 mice [10]. However, there has been no study using the metabolomics technology to analyze serum from the asthma model treated with BAW, to screen an alternative biomarker with anti-asthma effects.

As part of the investigation for other sensitive and reliable biomarkers representing anti-asthmatic effects, this study was designed to comparatively evaluate the serum biomarkers obtained from OVA+Vehicle and OVA+BAW treated BALB/c mice using the metabolomics-based proton (NMR) platform. Our results indicate that the metabolomics profile of serum collected from BAW treated asthma model could provide useful information to develop novel sensitive and reliable biomarkers for anti-asthma drugs.

## Materials and methods

### Preparation and analysis of BAW

BAW was prepared using the methods as described in a previous study [11]. Briefly, 20 g of freeze-dried *A. cochinchinensis* roots were ground to a powder, and a hot water extract was prepared by mixing with 1.2 L of deionized distilled water (dH_2_O), for 2.5 h in a hot water extraction device (Daewoong, Kyunggi, Korea). After completion of the aqueous extraction, the samples were filtered through Whatman No.2 filter paper (Whatman, Brentford, UK), following which they were evaporated in a rotary vacuum evaporator (EYELA, N-1100 series, Tokyo, Japan) and lyophilized. This freeze-dried, unfermented *A. cochinchinensis* root (UnFAR) powder was used for fermentation. The extraction yield in hot water was 60.7%.

The bacterial strains of *W. cibaria* used in the fermentation process were provided by Professor Hong Joo Son, Department of Life Science and Environmental Biochemistry, Pusan National University. To prepare the fermented products, UnFAR powder was dissolved in dH_2_O (pH 5.3) to 1% (w/v), and sterilized at 121°C for 15 min. After cooling to room temperature, *W. cibaria* precultivated in lactobacilli MRS broth (Difco Laboratories, Detroit, MI) to a final cell density of 10^7^ CFU/mL (OD_600_ = 0.1), were inoculated [5% (v/v)] to the UnFAR mixture solution. The mixture was then incubated in a shaking incubator (VS-8480, Vision Scientific, Bucheon, Korea) at 37°C and 150 x g for 4.3 days. The mixture solution fermented with *W. cibaria* was centrifuged at 12,000 x g for 10 min to obtain the fermented *A. cochinchinensis* products of *W. cibaria* (FARW).

To obtain the n-butanol fractions of FARW (BAW), an equal volume of butanol was added to the FARW. After vigorous mixing followed by incubation, the butanol phase was collected from each mixture by centrifuging at 12,000 x g for 10 min. Butanol extraction was repeated three times, after which all butanol phases were combined, evaporated under a rotary vacuum evaporator, freeze-dried, and stored at –20°C until further use. Finally, the collected BAW powder was dissolved in 0.5% Tween-20 solution in distilled water (dH_2_O) to the required concentration.

### Experimental design for animals

The animal protocols for this study were reviewed and approved for ethical and scientific care procedures by the Pusan National University-Institutional Animal Care and Use Committee (PNU-IACUC; Approval Number PNU-2015-0779). Six-week-old BALB/c mice (female) were purchased from Samtako BioKorea Co. (Osan, Korea). Before the animal experiment, the mice were given at least 1 week to adapt to the experimental condition. All mice were provided with ad libitum access to a standard irradiated chow diet (Samtako BioKorea Co.) and water throughout the experimental period. All mice were maintained in a specific pathogen-free (SPF) state under a strict light cycle (lights on at 08:00 h and off at 20:00 h) at 23 ± 2°C and 50 ± 10% relative humidity. BALB/c mice were housed at the Pusan National University-Laboratory Animal Resources Center, accredited by the Korea FDA in accordance with the Laboratory Animal Act (Accredited Unit Number: 000231) and AAALAC International according to the National Institutes of Health guidelines (Accredited Unit Number: 001525).

**Fig. 1:**
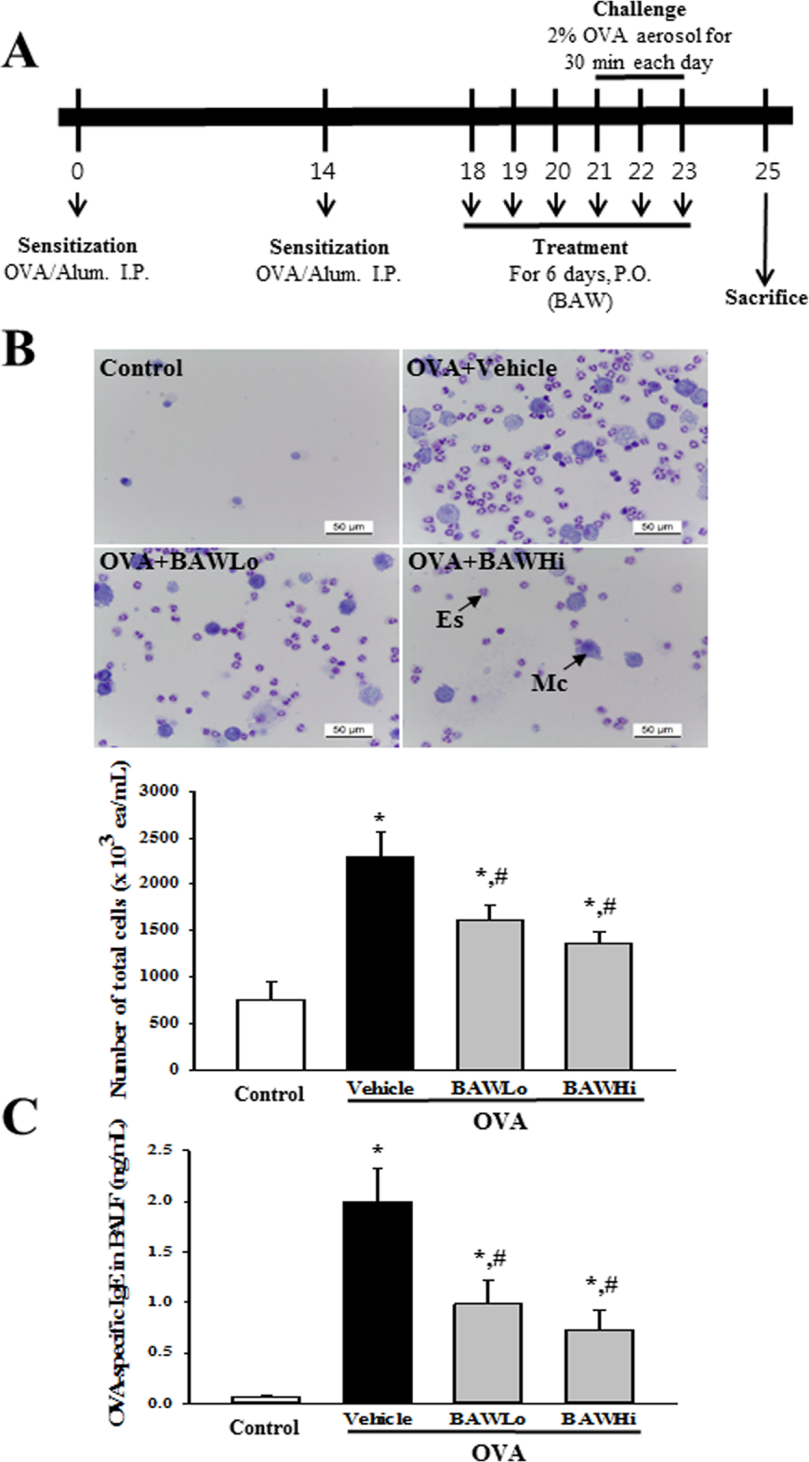
Number of immune cells and level of OVA-specific IgE in BALF. **a** Experimental scheme for OVA-induced asthma model and BAW treatment. **b** After the collection of BALF from the lungs, total cells were separated by centrifugation, and stained with May-Giemsa solution. Total cells, eosinophils and macrophages were measured within a 1 mm^2^ area under a light microscope at 400× magnification. **b** The concentration of OVA-specific IgE was quantified in BALF by an enzyme-linked immunosorbent assay (ELISA). The minimum detectable concentration of this kit is 20.7 pg/mL. The data shown represent the means ± SD of duplicates. *, *P* < 0.05 compared to the Control group. #, *P* < 0.05 compared to the OVA+Vehicle treated group. Abbreviation: Es, Eosinophil; Mc, Macrophage

The airway challenge of BALB/c mice was induced as previously described [12, 13]. Briefly, six-week-old BALB/c mice (female, *n* = 32) were randomly assigned to either Control group (*n* = 8) or OVA treated group (*n* = 24). The Control group remained an untreated condition during the experimental period. OVA-induced asthma was generated by sensitization for 20 days and challenge for 3 days. At day 1 and day 14, all mice were sensitized by intraperitoneal injection with OVA (20 μg) (albumin from chicken, Sigma-Aldrich; Merck KGaA, Darmstadt, Germany) emulsified with aluminum hydroxide (Alum, Sigma-Aldrich Co., St. Loius, MO, USA), in 200 μL of 1× PBS solution. At days 21–23, the sensitized mice were subjected to a 30 min airway challenge with 2% OVA in 1× PBS solution, administered by inhalation through a nebulizer (NE-C28, Omron, Tokyo, Japan). The OVA-induced asthma group was further divided into a Vehicle treated group (OVA+Vehicle, *n* = 8), low concentration BAW treated group (OVA+BAWLo, n = 8), and high concentration BAW treated group (OVA+BAWHi, n = 8). These groups were administered orally for 6 days from 3 days prior to the 1st challenge. The OVA+BAW treated groups received 250 and 500 mg/kg body weight of BAW, whereas the OVA+Vehicle group received orally the same volume of 0.5% Tween-20 solution. At 48 h after the final treatment, all animals were euthanized using CO_2_ gas, and tissue samples were acquired and stored in Eppendorf tubes at − 70°C until assay (Fig. 1a).

### Enumeration of total cells in BALF

After the BALB/c mice (*n* = 4) were anaesthetized with alfaxan (Alfaxalone®, Jurox Pty Ltd., Hunter Valley, Australia), BALF was obtained (yield: 80%, total volume of 0.8 ml) from mice of subset groups via tracheal cannulation using cold 1× PBS. After the centrifugation of BALF at 2000 x g for 5 min at 4°C, the supernatant were collected for ELISA analysis and the pellets were used for cell analysis. Total cells of BALF pellet were attached to slide glass using a cytospin (5 min, 500 x g, Hanil Electric, Wonju, Korea) and fixed in methanol for 30 s. These slides were processed in May-Grunwald solution (Sigma-Aldrich Co.) for 5 min, and subsequently in Giemsa solution (Sigma-Aldrich Co.) for 12 min. After rinsing with three times, the slides were covered, and then, immune cells were counted using the Leica Application Suite (Leica Microsystems, Wetzlar, Germany) at 400× magnification.

### Detection of OVA-specific IgE concentration in BALF

The OVA-specific IgE concentration in the BALF of mice (*n* = 4) was measured using an ELISA kit (BioLegend, San Diego, CA, USA), according to the manufacturer’s instructions. Briefly, wells were washed with washing solution (50 mM Tris, 0.14 M NaCl, 0.05% Tween 20, pH 8.0) four times, after which assay buffer (50 μL) and BALF (50 μL) were added to the wells coated with antibody; the plate was incubated for 2 h with shaking at room temperature. Next, the wells were washed with washing solution, 100 μL of detection antibody solution was added per well, and the samples were incubated for 1 h with shaking. After washing the wells, Avidin-HRP D solution (100 μL) was added to each well. The plates were then incubated at room temperature for 30 min, washed, and an enzyme reaction was initiated by adding the substrate solution; the plates were incubated in the dark at room temperature for 15 min. The reaction was terminated by adding 2 M H_2_SO_4_ solution, and the absorbance was measured at 450 nm using a Versa-max plate reader (Molecular Devices, San Jose, CA, USA).

### Histopathological analysis

To exactly measure the epithermal thickness of the bronchial tree, the histopathological features were analyzed in the same region of the lung. Briefly, the right lungs were collected from mice (*n* = 4) of a subset group, and fixed in 10% neutral buffered formalin for 48 h. The middle lobes were exactly trimmed from the fixed tissues, and embedded in the same direction and position to make paraffin blocks. After section the block into 4 μm thick slices, the lung section from #50 to #70 were collected from whole series of section. They stained with hematoxylin and eosin (H&E) (Sigma-Aldrich) and then the same region on the positioning of the bronchus within the bronchial tree microscopically examined (at 400× magnification) for identifying the infiltration of inflammatory cells into the peribronchial region. The epithelial thickness of the bronchial tube was also measured using the Leica Application Suite (Leica Microsystems).

### Metabolomics analysis

Appropriate serum samples (300 μL) obtained from mice (*n* = 4) of subset group were placed in microcentrifuge centrifuge tubes containing 300 μL D_2_O; 4 mM 3-(trimethylsilyl) propanesulfonic acid (TSP) was used as a qualitative standard for the chemical shift scale. After vortexing, serum samples were analyzed by NMR spectrometry within 48 h. All spectra were determined using a Varian Unity Inova 600 MHz spectrometer operating at a temperature of 26°C. The one-dimensional NMR spectra were acquired with the following acquisition parameters: spectral width 24,038.5 Hz, 7.55 min acquisition time, and 128 nt. Additional conditions of a relaxation delay time of 1 s and saturation power of 4 were set to suppress a massive water peak. NMR spectra were reduced to data using the program Chenomx NMR Suite (Version 4.6, Chenomx Inc., Edmonton, Alberta, Canada). The spectral region of δ0.0–10.0, excluding the water peak (δ4.5–5.0), was segmented into regions of 0.04 ppm, which provided 250 integrated regions in each NMR spectrum. This binning process endowed each segment with integral values, giving an intensity distribution of the whole spectrum with 250 variables prior to pattern recognition analysis.

All data were converted from the NMR suite Professional software format into the Microsoft Excel format (*.xls). One-dimensional NMR spectra data were imported into the SIMCA-P (Version 12.0, Umetrics Inc., Kinnelon, NJ, USA) for multivariate statistical analysis to examine the intrinsic variation in the data set. These data were scaled using centered scaling prior to principal component analysis (PCA) and partial least square-discriminant analysis (PLS-DA). With the scaling process, the average value of each variable is calculated and then subtracted from the data. Score plots of PCA and PLS-DA were used to interpret the intrinsic variation of the data. Variable importance plots (VIP) were also utilized to select putative metabolites related to OVA.

### Statistical analysis

Statistical significance was evaluated using one-way analysis of variance (ANOVA) (SPSS for Windows, Release 10.10, Standard Version, Chicago, IL, USA) followed by Tukey’s post hoc t-test for multiple comparisons. Data are presented as means±SD (standard deviation). *P* < 0.05 is considered to indicate a statistically significant difference.

## Results

### Anti-asthma effect of BAW in OVA-challenging model

**Fig. 2:**
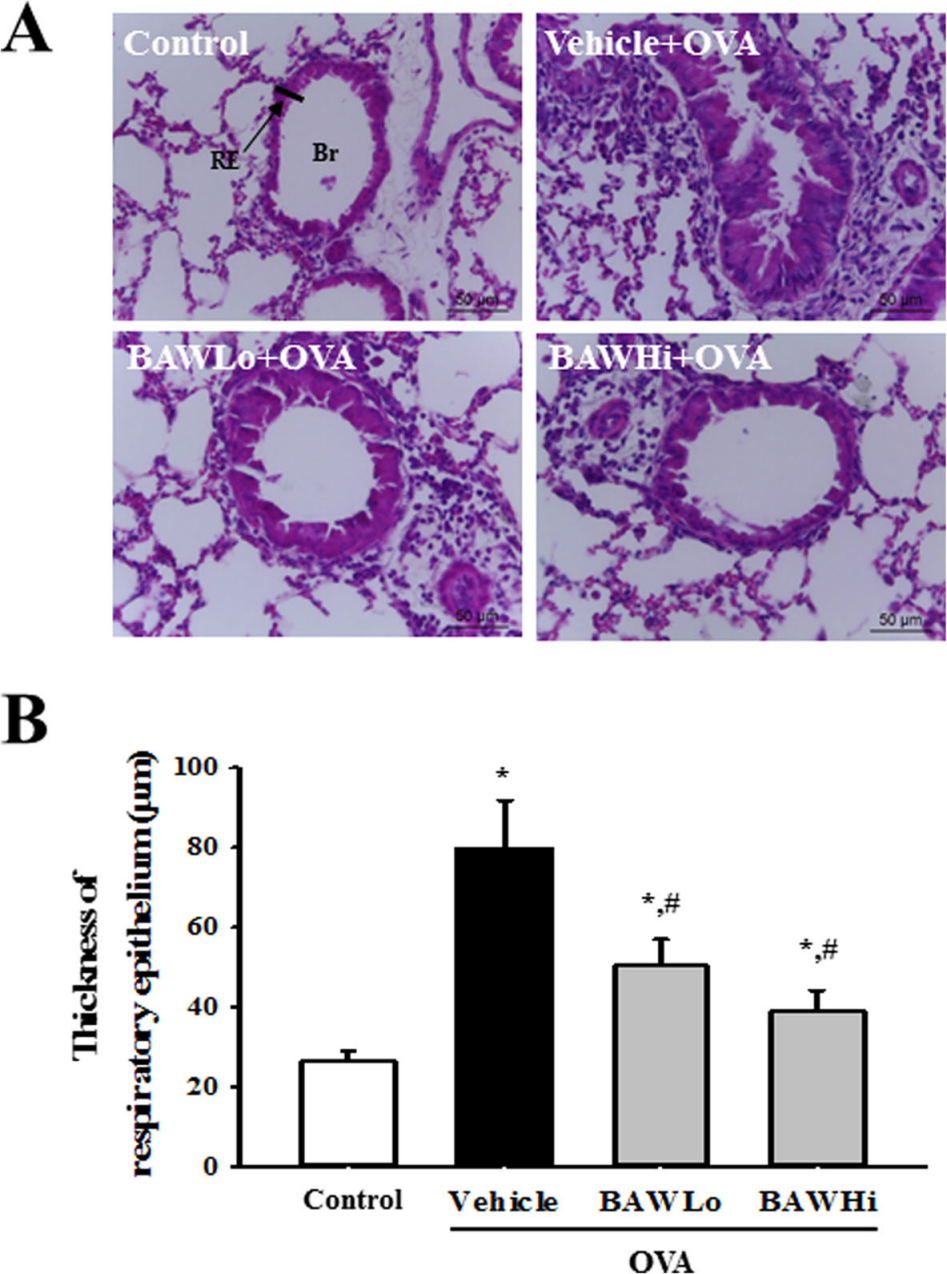
Histopathology in lung tissue. **a** The infiltration of inflammatory cells in the peribronchiolar region and the bronchial thickness were observed in H&E stained lung tissue at 400× magnification. Br, Bronchus; RE, Respiratory epithelium. **b** Thickness of respiratory epithelium was measured using the Leica Application Suite. The data shown represent the means ± SD of duplicates. *, *P* < 0.05 compared to the Control group. #, P < 0.05 compared to the OVA+Vehicle treated group

To confirm the anti-asthma effects of BAW in an OVA-challenged asthma mouse model, an alteration in the number of immune cells, IgE concentration and histopathological structure were measured in the Control, OVA+Vehicle and OVA+BAW treated BALB/c mice. We found that the total number of cells, eosinophils and macrophages in BALF were higher in the OVA+Vehicle treated group than in the Control group. However, the cell numbers were significantly decreased in the OVA+BAWLo and OVA+BAWHi when compared to the OVA+Vehicle treated group (Fig. 1a). A similar pattern was observed for the IgE concentration in BALF. The OVA-specific IgE levels were decreased in the OVA+BAWLo and OVA+BAWHi treated groups compared to the OVA+Vehicle treated group (Fig. 1b). Furthermore, a significant alteration was detected in the airway histological structure. The thickness of the respiratory epithelium and the infiltration of inflammatory cells in lung tissue were enhanced in mice that were sensitized with OVA (OVA+Vehicle treated group). These levels were found to be significantly decreased in the OVA+BAWLo and OVA+BAWHi treated groups (Fig. 2). These results suggest that BAW treatment improves the symptoms, including epithelial damages and infiltration of inflammatory cells, in the airways of the OVA-induced asthma model.

### Effects of BAW treatment on the metabolic profile of serum

**Fig. 3:**
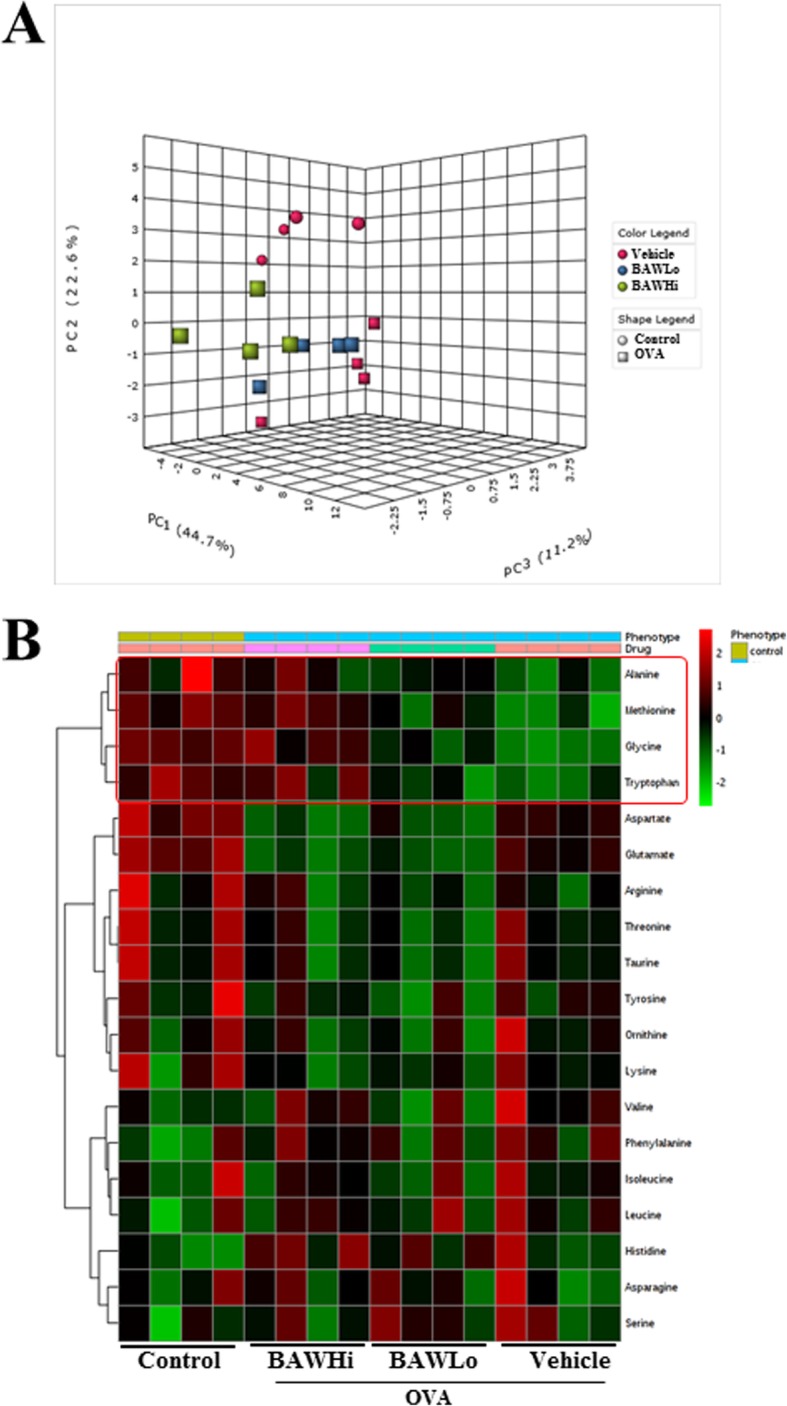
Spectral analysis of metabolomic pattern using OPLS-DA and heatmap. **a** OPLS-DA score plot obtained from NMR analysis comparing Control, OVA+Vehicle, OVA+BAWLo and OVA+BAWHi treated mice. **b** Heatmap shows significant fold level changes of metabolites in serum of all treated groups. Fold changes are derived from the fold differences over normalized means for each metabolite

**Fig. 4:**
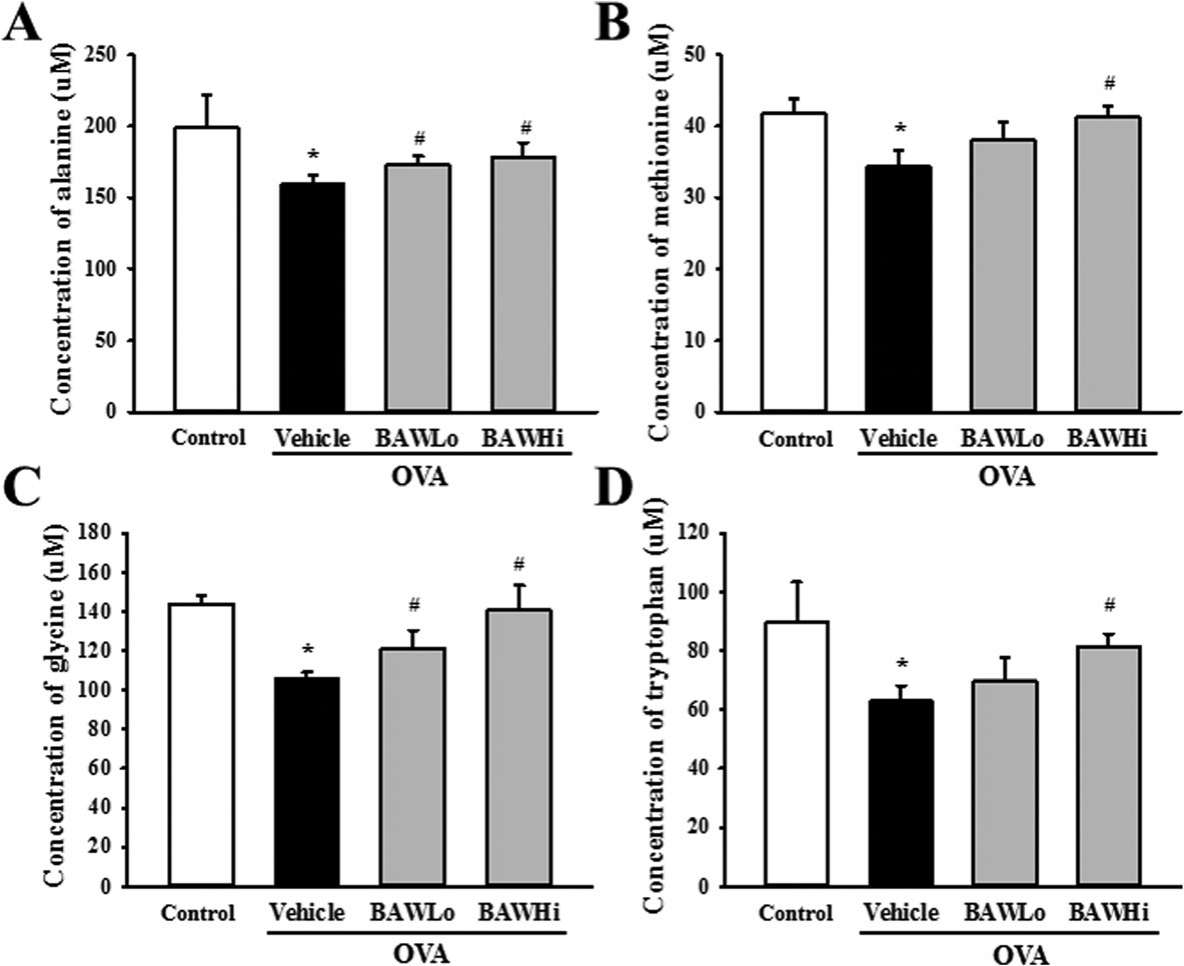
Concentration of 4 amino acids in serum after BAW administration. The data shown represent the means ± SD of duplicates. *, *P* < 0.05 compared to the Control group. #, *P* < 0.05 compared to the OVA+Vehicle treated group

To investigate whether BAW treatment affected the alteration on the endogenous metabolic profile of subset groups, the level of metabolites were analyzed in the serum of subset groups using ^1^H-NMR analyses. The pattern recognition using OPLS-DA score plots of the NMR spectra in metabolites profiling showed clear clustering between the Control group and the OVA treated group as well as the OVA+BAWLo and OVA+BAWHi treated groups from the OVA+Vehicle treated group. Most metabolites of OVA treated groups were clustered onto downstream of PC2 axis compared to Control group, while those of OVA+BAW treated groups were clustered onto middle area. Based on the OPLS-DA score plots, a total of 19 metabolites differed between the Control group and OVA+Vehicle treated groups were identified (Fig. 3a). Among these metabolites, 6 endogenous metabolites (Serine, Histidine, Valine, Phenylalanine, Isoleucine and Leucine) were increased and 13 metabolites (Aspartate, Glutamate, Asparagine, Glycine, Arginine, Threonine, Taurine, Alanine, Tyrosine, Methionine, Tryptophan, Ornithine and Lysine) were decreased after the treatment of OVA. However, only 4 metabolites were significantly recovered in OVA+BAW treated group. Therefore, we selected to investigate the effects of BAW treatment using heatmap (Fig. 3b). Particularly notable was the detection of 4 metabolites in response to BAW treatment, while other metabolites were maintained constant level regardless of BAW treatment. These notable metabolites were categorized into an amino acid group containing alanine, glycine, methionine and tryptophan. In detail spectra analyses, the OVA+Vehicle treated group showed a significant decrease with 19.7% (alanine), 26.6% (glycine), 17.1% (methionine) and 29.2% (tryptophan) on the concentration of four metabolites relative to those of Control group. However, these levels were significantly recovery with 8.2 and 12.0% (alanine), 15.2 and 33.3% (glycine), 11.8 and 20.6% (methionine), and 11.1 and 28.6% (tryptophan) after OVA+BAWLo and OVA+BAWHi co-treatment (Fig. 4). These results suggest that BAW treatment results in a recovery of 4 amino acids (alanine, glycine, methionine and tryptophan) related to asthma.

## Discussion

Metabolomics is a highly sensitive analytical tool with capability to simultaneously detect numerous metabolic markers in a single sample [14]. This approach has been applied in several studies to analyze the BALF or serum of OVA-induced asthma model, as well as asthma model treated with therapeutic drugs [7–10]. However, to date, there are no metabolomic studies that have investigated these metabolic changes in the serum of OVA-induced asthma mice treated with BAW. The result of the present study therefore provides important information for a metabolic profile, offering clues for a predictable anti-asthmatic marker.

Meanwhile, BAW used in this study was reported to have anti-inflammatory and anti-asthma effects. Inflammatory responses significantly suppressed with BAW treatment through differential regulation of the iNOS-mediated COX-2 induction pathway and inflammatory cytokine expressions in LPS-activated RAW264.7 [11]. Treatment with BAW decreased the infiltration of inflammatory cells, bronchial thickness, serum concentration of OVA-specific IgE and expression of Th2 cytokines, while a significant recovery was observed on the goblet cell hyperplasia, MMP-9 expression, VEGF signaling pathway, acetylcholine esterase (AChE) activity and muscarinic acetylcholine receptor (mAChR) M3 downstream signaling pathway in an ovalbumin (OVA)-induced asthma model after treatment with BAW [15]. These effect of BAW reached the maximum level in OVA-induced BALB/c mice treated with 500 mg/kg and that these effects can last for 48 h [16]. Based on above results, we selected BAW as anti-asthma product to identify novel biomarkers that can predict the therapeutic effects for asthma and determined two different doses of BAW (250 and 500 mg/kg).

**Fig. 5:**
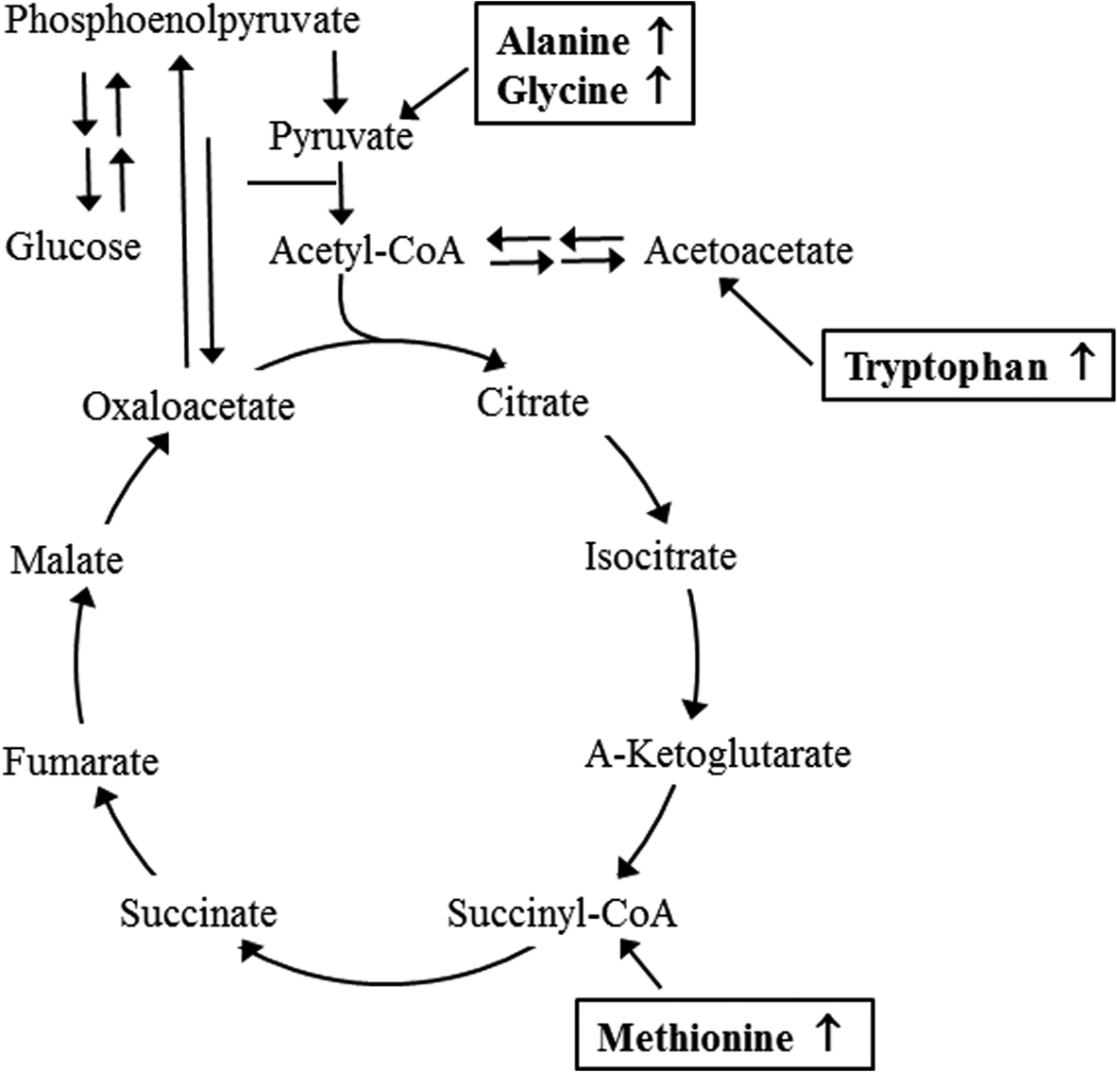
Metabolic pathway related to the 4 amino acids. Among the various metabolites in TCA cycle, the levels of 4 amino acids altered after BAW treatment are indicated in the box

An alteration on metabolic profile in serum and BALF sample of asthma model have been investigated in BALB/c, A/J and C57BL/6 mice. Significant changes in 13 metabolites related with lipid, amino acid and energy metabolism in the BALF of OVA-induced BLAB/c mice. Of these, only 11 metabolites (choline, cholic acid, cortol, cholesterol, phosphatidylcholines, triglycerisdes, 5-methoxy-tryptophan, creatine, mannose, galactose and arabinose) were dramatically recovered after Dex treatment [8]. Also, a significant alteration on 19 metabolite concentrations were detected in the serum of OVA-challenged BALB/c mice, although only 5 metabolites of these 14 metabolites (arginine, C3DC1C4OH, carnitine, serotonin, and tyrosine) recovered in OVA+Fenretinide (FEN) treated mice [9]. Furthermore, 6 metabolites related with sphingolipid, bile acid, purine and fatty acid metabolism were altered in the serum of OVA-induced asthma model: dodecanoic acid (P1), myristic acid (P2), phytosphingosine (P3), sphinganine (P4), inosine (P13) and taurocholic acid (P15) [7]. Moreover, in OVA- challenged C57BL/6 mice treated with the omega-3 fatty acid eicosapentaenoic acid (EPA), the metabolomic profile analysis of the lung revealed that 12-OH-17,18-EpETE can be considered as one of the major molecules amplified by EPA administration and allergic inflammation [10]. In the current study, we investigated the profile of endogenous metabolites in serum of asthma mice treated with BAW, to characterize putative biomarkers for anti-asthma effects. The notable observation was the dramatic recovery of 4 amino acids related to the tricarboxylic acid (TCA) cycle in the OVA+BAW treated group compared with OVA+Vehicle treated group (Fig. 5). However, this result differed from the previous four studies, although some amino acids have been indicated as markers for anti-asthmatic effects. The variation of our results and those of previous studies can be attributed to the compositional properties of the therapeutic drugs, sample for analysis, and genetic background of mice. Hence, more research is required to understand which component directly correlates with the four amino acid metabolites.

Only several studies have provided some evidences for the correlation between some metabolites and anti-asthma effects. Alanine was detected as significant metabolites in exhaled breath condensate of mild asthmatic patients and urinary metabolic composition of asthma exacerbation [17, 18]. The serum level of methionine was remarkably increased in asthma patients during H-NMR-based metabolite profiling [19]. Also, the activity of indoleamine 2,3-dioxygenase-1 (IDO), an index of tryptophan breakdown, was decreased in airway of children with asthma [20]. Therefore, these results from previous studies support the reliability of metabolites identified by present study although additional studies will be need.

## Conclusions

Overall, the present research is the first study to examine the metabolic changes in BALB/c mice treated with OVA+BAW, and to correlate the changes in specific metabolites with the anti-asthmatic effects of BAW. In addition, the results presented herein provide evidence that 4 amino acids could be considered as biomarkers for predicting anti-asthma effects induced by natural fermented products therapy.

## Data Availability

Available.

## Abbreviations

^1^H-NMR: ^1^H nuclear magnetic resonance
BALF: bronchoalveolar lavage fluid
BAW: butanol extract of *Asparagus cochinchinensis* roots fermented with *Weissella cibaria*
EPA: eicosapentaenoic acid
FARW: fermented *A. cochinchinensis* products of *W. cibaria*
H&E: hematoxylin and eosin
OVA: ovalbumin
PCA: principal component analysis
PLS-DA: partial least square-discriminant analysis
Th2: T helper 2
UnFAR: unfermented *A. cochinchinensis* root
VIP: variable importance plots
